# The Role of Intersectoral Action in Response to COVID-19: A Qualitative Study of the Roles of Academia and the Private Sector in Colombia

**DOI:** 10.34172/ijhpm.2021.100

**Published:** 2021-08-30

**Authors:** Simon Turner, Ana María Ulloa, Natalia Niño, Vivian Valencia Godoy

**Affiliations:** ^1^School of Management, University of Los Andes, Bogotá, Colombia.; ^2^School of Medicine, University of Los Andes, Bogotá, Colombia.

**Keywords:** Intersectoral Coordination, COVID-19, Academia, Private Sector, Colombia

## Abstract

**Background:** The integration of health services with other sectors is hypothesised to support adaptation of health systems in response to coronavirus disease 2019 (COVID-19). This study identified barriers and enablers associated with intersectoral coordination at an early stage of the pandemic. The study focused on the roles played by the academic and private sector in different areas of public health planning and delivery concerning COVID-19 in Colombia.

**Methods:** A qualitative approach was used to understand stakeholders’ experiences and perceptions of intersectoral working in response to COVID-19 in three Colombian cities (Bogotá, Cali and Cartagena). Between March and November 2020, data was collected via semi-structured interviews conducted online with 42 key actors, including representatives of governmental bodies, universities, and professional associations. The dataset was analysed thematically using a combination of inductive and deductive methods.

**Results:** Organizations adjacent to the health system, including universities and the private sector, supported responses to COVID-19 by providing evidence to inform decision-making, additional service capacity, and supporting coordination (eg, convening intersectoral "roundtables"). The academic and private sector involvement in intersectoral coordination was stimulated by solidarity (being the "right thing to do") and motivation for supporting local companies (reopening the economy). Intersectoral working was influenced by pre-existing (substantive) and emerging (situational) enablers and barriers.

**Conclusion:** This study showed that intersectoral coordination has played an important role in responding to COVID-19 in Colombia. Coordination was influenced by substantive and situational enablers and barriers. Based on our findings, policy-makers should focus on addressing substantive barriers to coordination, including the pre-existing tensions and mistrust among national and local healthcare actors, strict regulations and limited financial and human resources, while providing support for situational enablers, including alignment of public and private actors’ interests, intersectoral government support and establishing frequent communication channels and formal spaces of interaction among sector, in processes of decision-making.

## Background

**Key Messages**

** Implications for policy makers**

- National authorities in Colombia should focus on supporting the formal participation of universities and the private sector in future public health planning processes in Colombia, eg, by investing in infrastructure for supporting intersectoral collaboration.
- Coronavirus disease 2019 (COVID-19) stimulated intersectoral collaboration in Colombia by encouraging common purpose and alignment of interests among health system actors, universities, and private enterprise in the cities of Bogotá, Cali and Cartagena. This experience has potential lessons for future management of COVID-19 and/or other contagious disease outbreaks.
- Internationally, health system leaders should invest in substantive or pre-existing resources, including the nurturing of intersectoral relationships and trust, which can be mobilized quickly as situational or emergent resources in response to crises like COVID-19.

** Implications for the public**

This qualitative study explored experiences of responding to coronavirus disease 2019 (COVID-19) through interviews with participants representing healthcare, universities, and the private sector in Colombia. In particular, the study examined perceived barriers and facilitators to productive relationships among these sectors to respond in a collective way to COVID-19. Collaboration between different sectors, including health services, universities, and private enterprise, was found to support the response to COVID-19 in the Colombian cities of Bogotá, Cali and Cartagena. Within Colombia, there is a need for public investment to deepen the relationships between these sectors which can then be drawn upon to aid the continuing response to COVID-19 and future pandemics.

Coronavirus disease 2019 (COVID-19) has generated major challenges that surpassed the preparations for a pandemic by healthcare systems worldwide. Collaboration across all sectors of society has been advocated globally as a necessary response to the pandemic.^1^ According to World Health Organization (WHO) guidance,^2^ it is recommended that strengths from across all sectors, and communities, are united to contain the disease and reduce its societal impact. The sectors most commonly addressed are ministries of education, employment, housing, infrastructure, and social sector, as well as private and nongovernmental actors. Furthermore, early evidence on responses to COVID-19 from Asian countries indicated that, to manage this public health crisis, multisector participation is essential and that coordination of health services with other sectors facilitates the response of the health system to adapt to the system “shock” represented by the virus.^3^

 The aim of this paper is to analyse how intersectoral coordination took place in three Colombian cities (Bogotá, Cali and Cartagena), describing the main roles that two sectors, academic institutions and private enterprise, assumed in their efforts to assist the response of the health sector to COVID-19. Evidence for this paper is derived from a qualitative study which analysed responses within Colombia to the COVID-19 pandemic using three in-depth case studies.^4^ In the next section, we describe the need for intersectoral coordination in the health sector, and its calling under the context of COVID-19 in Colombia. Then, the methods used to study coordinated responses to COVID-19 are outlined. This is followed by findings focused on barriers and enablers associated with intersectoral coordination. Finally, the discussion section summarises the main insights from the study and their implications for research, policy, and practice.

###  COVID-19 and the Call for Intersectoral Coordination

 For many decades, the WHO has promoted and recognized health as the product of all functions of society.^5^ It has proposed intersectoral collaboration, or promoting relationships between sectors rather than each sector acting alone, as a promising strategy to tackle some of the major public health problems associated with unequal access to care.^6,7^ This “Health in All Policies” approach, as it is sometimes called, stresses that to address social determinants of health, work across different policy fields is necessary. For this reason, more attention has been placed on intersectoral work in policy-making rather than as an outcome of practice involving government and non-government actors.^8^ This study focusses on mechanisms of intersectoral coordination between government, academia, and private enterprises, rather than inter-government agency coordination to support healthcare, to which another literature applies, including vertical relationships between national and local agencies^9^ and horizontal relationships across agencies.^10^ In the analysis for a related paper, we are focussing on inter-agency coordination within the health system in response to COVID-19 within Colombia.

 There is an extensive literature on intersectoral coordination, written from different conceptual perspectives and aspects of public health that such coordination aims to improve. One conceptual distinction concerns the scale of analysis of coordination. One approach emphasises system level coordination, or how actors from different sectors may contribute to planning at the macro level, as the following definition highlights: “a deliberative, recognized, purposeful relationship constituted of government, non-government, not for profit, business, academia, communities, policy-makers, managers, clinicians and multi-organizational stakeholders at the local, national and global scale, along and across horizontal and vertical axes.”^11^ Another perspective stresses the interpersonal or micro level, defining intersectoral collaboration as a “negotiation between people from different organizations with a commitment to working together to secure improvements which could not have been achieved by acting alone.”^12^

 Different purposes of intersectoral collaboration have also been studied. For example, in relation to improving health equity, there is no consensus on what real collaboration entails, nor have many studies examined how intersectoral collaboration around health systems occurs in practice.^11^ Within this extensive literature on intersectoral collaboration, we focused on a limited and responsive form of intersectoral collaboration to support the responsive planning and delivery of health services in response to the specific health issue of COVID-19. COVID-19 represents a particular context for studying intersectoral coordination. The distinction between “opportunity” and “problem” gaps highlights this context. Some of the existing literature could be seen to advocate intersectoral coordination to meet an “opportunity gap” (eg, improving health equity), while emphasising such coordination in response to COVID-19 appears to address a “problem gap,” that is, an acute and pressing challenge with a novel cause that has received urgent attention from health systems worldwide. Calls for, and practices of, intersectoral collaboration in response to both gaps are important in their own right, but are likely to be characterised differently. For example, intersectoral collaboration in responding to COVID-19, a novel, highly transmissible and potentially lethal contagious disease, is likely to be different to intersectoral collaboration intended to respond to other contagious diseases or to non-communicable diseases. Our approach only considered intersectoral collaboration that assisted and supported health service responses to COVID-19, but did not examine the potential for intersectoral collaboration to tackle the broader social and economic impacts of COVID-19.

 The emergence of the COVID-19 pandemic has been accompanied by a renewed emphasis on intersectoral coordination to augment health system capacity and acquire access to additional expertise and innovation.^2^ However, encouraging intersectoral coordination to support the response, implies resolving underlying organizational coordination problems that have thwarted coordination historically. Little is known about how health systems have approached and sought to overcome such coordination problems to make rapid adaptations to health systems to respond to COVID-19. Our findings explore the ways in which intersectoral coordination in relation to COVID-19-related public health tasks were planned and implemented across different Colombian cities, including analysis of barriers and enablers to working across intersectoral boundaries.

 In Colombia, following processes of reform and decentralization implemented in Latin America in the health sector in the 1990s, activities in public health have continued to suffer from scant human, physical and financial resources, as well as little support from other sectors. Departmental and municipal entities, in charge of managing primary care and public health actions, have had difficulties implementing an intersectoral approach to health, despite its promotion by the Ministry of Health and Protection with the creation of an intersectoral committee in 2011.^13^ A lot of the responsibility in public health surveillance, control, and intervention has traditionally fallen exclusively under the head of departmental and local secretariats of health and their network of public hospitals.^14^

 The pandemic caused by the coronavirus changed, at least momentarily, this situation. As in other countries, intersectoral action in the country was called for and expected from the beginning of the crisis. In January 2020, before any registered cases of COVID-19, Colombia’s Ministry of Health and Social Protection organized an intersectoral committee, involving institutions such as Migration Colombia, transport authorities, and the national institute for health, to plan for control and mitigation actions. Intersectoral coordination was listed as a priority in the emergency action plan.^15^ However, this action did not only occur within government agencies but saw the participation of new actors such as academia and the public sector as this study reveals. Recognition of striking socio-economic, humanitarian and health impacts to come with the pandemic and lockdown measures, left a fertile ground for seeking innovative intersectoral action.

## Methods

###  Study Design 

 The study followed a case study approach^16^ to understand, from the perspectives of stakeholders involved in intersectoral planning and delivery of health services, the responses of three Colombian cities to COVID-19 between March and November 2020.

###  Case Selection 

 Colombia was chosen as a case study for examining intersectoral coordination because it has been severely affected by COVID-19, reporting the second highest number of cases and deaths in South America after Brazil, and the eleventh highest internationally.^17^ Colombia was also chosen as an upper-middle-income economy, that faced resource pressures and technical constraints in relation to health services prior to the pandemic´s onset, making it an example of the challenges faced by less developed economies in responding to COVID-19.^18-20^

 In response to COVID-19, 50% of hospital capacity was allocated to the treatment of COVID-19 patients nationally.^21^ Our study focused on intersectoral coordination within three cities, Bogotá, Cali, and Cartagena which have consistently presented a high number of COVID-19 cases relative to the rest of Colombia (see Table 1 for overview of cities´ demographics and health systems). We recognise as a limitation that our study only focused on urban centres; the findings may not be applicable to smaller cities and/or rural areas of Colombia, where the presence of academia and private sector actors may be more limited, or absent. Further research should examine barriers and facilitators to intersectoral collaboration in such settings.

**Table 1:** Overview of Cities’ Demographics and Health Systems

		**Case Studies **	
		**Bogota **	**Cali **	**Cartagena **
Health insurance coverage	Population^22^	7.9 million people	2.3 million people	1.1 million people
	Health insurance^23^	Subsidised regime covers 1 470 319 (18.42%) Contributory regime covers 6 378 456 (79.91%) Exception and special regime represents 1.66%	Subsidised regime covers 753 825 (31.74%) Contributory regime covers 1 590 179 (66.96%) Exception and special regime represent 1.29%	Subsidised regime covers 580 165 (50.20%) Contributory regime covers 552 521 (47.81%) Exception and special regime represent 1.98%
Local health services	Delivery of health service	Four geographical sub-networks lead the delivery and planning of public health services in the city.	Five ESE that operate in Cali and hold a network of hospitals, health centres and health posts.	Three subnetworks.
	Installed hospital Capacity^24^	7157 hospital beds 450 intermediate care units 2384 intensive care units	3142 hospital beds 306 intermediate care units 848 intensive care units	1241 hospital beds 197 intermediate care units 360 intensive care units
Policy responses to COVID-19	First case detected	March 6, 2020	March 15, 2020	March 8, 2020
	ICU Management (primary data)	The city through the regulatory centre for emergencies assumed the administration of all ICUs across the private and public sector.	The local government with a national regulatory centre for emergencies took control of ICUs through "solidarity bag" strategy.	Cartagena did not assume the administration of ICUs because the affectation level by COVID-19 was moderate.
	Intersectoral participation (primary data)	Intersectoral committees were established with local authorities, universities and private sector.		Weekly roundtables meetings were introduced with the participation of intersectoral stakeholders to coordinate the local response to COVID-19.
	Expansion of intensive care beds^25-27^	935 to 2384	749 to 848	200 to 360
	Number of university labs per city^27^	7	1	2
Affectation level by COVID-19	Low, moderate and high affectation^28^	High	High	Moderate

Abbreviations: ESE, Spanish acronym for State Social Enterprises; COVID-19, coronavirus disease 2019; ICU, intensive care unit.

###  Research Participants 

 Semi-structured interviews were conducted between June and November 2020 in the cities of Bogotá, Cali, Cartagena, and nationally (Table 2). Study participants included stakeholders representing health-related governmental bodies (eg, ministries, health institutes and health secretariats); academia (public and private universities); and private sector actors (covering (*a*) business associations representing private enterprise and (*b*) not-for-profit, non-governmental organizations including development agencies and chambers of commerce in each city that were identified through documentary analysis to be of relevance in the response to COVID-19). The study therefore focussed on intersectoral collaboration between health-related governmental bodies, academia, and private sector actors. The paper did not focus on collaboration among different types of governmental organization (eg, between health agencies and those government bodies responsible for migration and transport) because our particular interest was in collaboration among organizations from different economic sectors (government, academia, private enterprise), including their differing motivations to respond to COVID-19 and barriers and facilitators to collaboration.

**Table 2:** General Characteristics of the Interviewees

**Type of Organization **	**National Level**	**Bogotá**	**Cali**	**Cartagena**	**Total (N = 42)**
Governmental body	4	3	5	9	21
Private sector organizations	2	2	2	3	9
Academic sector	-	4	6	2	12

###  Sampling 

 Organizations that played a role in intersectoral coordination in response to COVID-19 were selected. Purposive sampling of such organizations was informed by searching documentary evidence (eg, newspaper reports and policy reports), contact information for interviewees was identified by searching organization’s websites and through snowball sampling. Senior informants that could provide an overview of their organization’s experiences of COVID-19 were prioritised. The sample size reflected data saturation whereby no added information from interviewees’ perspectives emerged.

 As data collection took place during the Colombian lockdown, interviews were conducted virtually using online platforms such as Teams, Zoom and Google Meet. Interviewees provided informed consent. The interviews were conducted in Spanish, informed by a topic guide, audio-recorded and professionally transcribed. Relevant interview quotations were translated into English by bilingual researchers.

###  Inclusion and Exclusion Criteria 

 The inclusion criteria of this study included key actors that held a senior-level position within government entities, private and public universities, private guilds and associations such as chambers of commerce, not-profit organizations and representatives of business organizations at local and national level, that were part of intersectoral work in response to COVID-19, in the three cases. Staff holding middle and low-level positions were excluded.

###  Data Analysis 

 The research team read the interviews collectively and analysed them thematically using a combination of inductive and deductive methods.^29^ The interview data were coded using New NVivo software. We developed 32 codes that described organizational responses to the pandemic. To examine inter-organizational coordination, we explored four of these codes in-depth: coordination, involvement of other sectors, public and private relationships, and academia’s role.

## Results

 The results focus on the partnering roles of academia and private enterprise in response to COVID-19 in Colombia. For each actor, we describe their main roles in their response to the pandemic and perceived barriers and facilitators to intersectoral actions in response to COVID-19.

###  Academia 

####  Roles

 The academic sector fulfilled three main roles in support of the local health system: (1) advisory (providing evidence to support decision-making), (2) infrastructure (provision of lab services for COVID-19 testing), and (3) networking capabilities (building an infrastructure for undertaking COVID-19 related research) (Table 3).

**Table 3:** Roles of Academic Institutions

**Role**	**Actions**	**Outcome**	**Examples **
Advisory	Provision of evidence Data analysis Consulting	Support local authorities with decision-making Participation in committees and town halls Opinion pieces and science communication	Georeferencing and epidemiological models contributed to identification of hot areas and case distribution (Cali) A committee of health experts from the different universities in Cali and hospitals and clinics (COPESA) was formed in March 2020. It was led by the ex-governor of the department, a medical doctor and important political figure in the region (Cali) Predictive mathematical models supported lockdown and mobility measures (Bogotá) Agreement between health secretariat and private university to provide ongoing analysis of the situation in the city, considering public health measures implemented (Cartagena)
Infrastructure	Provision of laboratory services for COVID-19 testing	Support national network of laboratories at the beginning of the pandemic Facilitate timely diagnosis and tracing of positive cases	Academic laboratories today represent 18.51% (30 facilities) of the total national network. There are 7 laboratories from universities in Bogotá, 2 in Cali, and 2 in Cartagena A private university led a project to provide 100 000 free PCR tests for the detection of coronavirus to taxi drivers, bus drivers, fire fighters, delivery personnel, etc (Bogotá)
Networking	COVID-19 research development Partnerships with industry	Support with development of research and manufacture of medical supplies	A private university built a partnership with a domestic appliances company and public entity to produce mechanical ventilators that are still waiting for approval (Bogotá)

Abbreviations: COVID-19, coronavirus disease 2019; COPESA, Comité Público Privado de Expertos en Salud; PCR, polymerase chain reaction.

####  Coordination Enablers and Barriers 

 The intersectoral coordination of universities and local health systems was propelled by scientific and policy uncertainty concerning the virus’ behaviour and appropriate intervention measures. First, the pressure for timely data and epidemiological evidence, and the lack of scientific consensus about the virus and the disease, was an enabler for collaboration between academia and local health secretariats, including recognition that inviting “ideas” and “opinions” was necessary where rigorously produced evidence was lacking:

 “*Slowly the pandemic synchronised people around the fundamental issues. That creates a collaborative environment or at least the desire to collaborate. Ideas are heard, proposals are seen, possibilities open. Scientific rigour was surpassed, not because it is bad*,* but because under these circumstances we have learnt that socializing information and inviting people to have an opinion is something positive. We don’t have scientific evidence that can guarantee that plan ‘A’ is the right one, and ‘B’ is not” *(Secretariat of health representative, Bogotá, SH-B-002).

 According to various stakeholders the crisis motivated intersectoral action and facilitated dialogue and mutual understanding between the academy and local authorities.

*“If were not in a crisis it would be hard to think how to show this knowledge we are producing to the mayor, for instance. I would have to go and tell the principal of the university to talk to her. But, in this situation, it is evident how everyone’s ears and understanding is open” *(Leader of a laboratory of a private university, Bogotá, SH-B-024).

 Second, formal spaces of interaction, such as periodic committees, were developed within secretariats of health in all cities studied to facilitate decision-making and collaboration with academics. In the case of Bogotá, the secretariat of health invited individuals from five universities to participate in the committees of experts that were advising the local government on mitigating COVID-19:

*“We worked with groups of scholars (…) all of them with diverse academic points of view. They were part of the committees; with them we began thinking what was best to face the pandemic. The Secretariat of Health and the town hall opened these spaces for discussion, so the mayor and the secretary of health could make the best decisions in terms of policy” *(Health Secretariat Representative, Bogotá, SH-B-025).

 Similarly, in Cali, individuals employed by the schools of public health, collaborated with the development of strategies and models for epidemiological surveillance that were used by the local secretariat of health. In Cartagena, the health secretariat relied on a local private university to give scientific and technical support to public health planning across the city.

 Third, comparing the experiences of different universities in setting up their research laboratories for diagnosis, it is important to consider pre-existing conditions as enablers. The first of these was universities’ capabilities in mobilising external resources to invest in developing their testing capacity, on top of their established infrastructure. This included external fundraising strategies for acquiring finance from individual donors and from the private sector (a university in Bogotá, for example, reached US$1.7 million in donations). Fundraising, especially among privately owned universities, helped to overcome the financial strain of achieving laboratory certification. This made some universities better placed than others in how they could quickly respond to the crisis and adapt their research agenda. The head of a molecular biology laboratory of a private university stressed this fact:

*“The great advantage we have is the infrastructure we had as university. We have an emphasis in molecular biology, so we had trained students on molecular biology techniques; also, the quality of our administrative personnel; the quality of our infrastructure, we were prepared with the necessary equipment” *(Leader of a laboratory of a private university, Bogotá, SH-B-024).

 The second enabler of this type is pre-existing and informal relationships utilised by some academic staff. In our interviews we heard common stories among people from academic laboratories that had to resort to friends and close colleagues to source important supplies where these were missing. Challenges of obtaining supplies, due to high demand locally and internationally, were sometimes overcome through informal networks of cooperation among laboratories. These networks among colleagues also worked for the formation of committees of experts advising the health secretariats. However, while informal collaboration took place among long-standing colleagues, there appeared to be further potential for developing organized and durable intersectoral ties among different organizations and providing opportunities for new actors from the university sector to participate.

 In terms of barriers that were more specific to the context of the pandemic we found constraints related to time and resources. The first barrier, as described by some participants of the study, had to do with clashes between the timelines for academic research and those of policy decision-making. COVID-19 reached Colombia in the first week of March 2020, and, as in other countries, local health systems were unprepared and needed urgent actions. For this reason, an interviewee working in academia in Bogotá felt that, to be able to collaborate with decision makers in the health sector, they had to conduct research that could show results in a brief period. Differences in timelines (for research and decision-making) and difficulties balancing expectations for the applicability of research outcomes were identified as challenges.

 This interviewee also emphasized that the emergency had triggered the need to provide wide-ranging evidence, and collaborate with peers from other disciplines, to generate the types of recommendations desired by policy-makers for decision-making (eg, georeferencing for identification of “hot areas”) in diverse topics of relevance that included research about the virus, the disease, the lockdowns, epidemiological surveillance implementation, transportation, planning and delivery, among many other topics. Both the speed and breath of research that were demanded from academics were sometimes felt to run against usual academic standards, particularly those around the timeframe typically needed for delivering robust research findings and implications. But many were willing to do rapid research and data collection given the conditions of urgency and crisis.

*“From the academic perspective, you are used to processes that are completely different from the processes of the Secretariat. We had to understand their dynamics. The university was always opened to do so, that made it easier that we could get closer. We understood the needs of governmental institutions, and we also found ways to share our knowledge and our expertise. (…) At the beginning of the pandemic a lot of people were willing to contribute, moved by the need of giving knowledge to try to provide solutions quickly*”(Lecturer in Public Health, Cali, SH-C-005).

 Some universities provided slack to support responsive work on COVID-19 by liberating academics from other responsibilities to support the health secretariats, setting up internal research funding, and making visible and public the work of its professors. In other universities, researchers had to juggle their existing research commitments and burgeoning teaching load with the new priority of COVID 19-related research, including participation in a wave of external calls for research funding applications.

 The second barrier was perceived resource constraints. COVID-19 placed a financial burden on universities and research centres (eg, due to the risk of falling student numbers and costs associated with transitioning to virtual education), affecting the roles played by universities in response to the pandemic. This was on top of a historically precarious situation of the development and financing of science in Colombia.^30^ With limited resources to contribute to the COVID-19 research agenda, some research groups did not feel, on occasion, that they possessed sufficient personnel to attend to the demands of local authorities or vice versa.

*“I am a professor from a public university in Cali, but up to this moment I have not received any monetary fees from the university to support Cali’s Health Secretariat, neither do I have a contract with the Secretariat. I´ve been working three months, 16 hours a day, only for love to my craft and because is the right thing to do” *(Academic, public university, Cali, SH-C-003).

 Laboratories from universities that turn to diagnosis also experienced challenges with resources, affecting their coordination with health secretariats. Some laboratories had periods in which they struggled to have constant access to the necessary supplies to process the samples such as reagents and lab pipette tips. Some laboratories expressed that the secretariats of health had agreed to provide supplies such as the reagents, however this took longer than expected; for this reason, they had to delay opening while others had to find additional resources to do their work or recur to other colleagues.

*“Just when we had the laboratory ready (…) We hired bacteriologists so we could do the job, but we did not have the supplies to work, we did not have the reagents (…) We did not have the resources to fund something that is so expensive, and we already had made several investments. We did not have the money to buy the diagnostic kits. They never sent us a thing. We sent letters to [a national institute] saying “we are here ready to work we just need the supplies to work” they just replied saying, please wait. Still, things never came” *(Leader of a laboratory of a public university, Cartagena, SH-D-024).

 A third barrier reported, particularly for the case of laboratories, was complying with the requirements to transform a laboratory for research into a certified laboratory for COVID-19 diagnosis. While participating universities had a robust infrastructure to run molecular biology tests, most of the leaders of laboratories interviewed stressed that becoming a certified laboratory implied significant administrative work, the need of training additional human resources and substantial financial investment. This barrier was particularly stressed by participants working in public universities. For example:

*“So, we enrolled ourselves on that race, the race of creating a laboratory. It was a painful process, we had to learn everything (…) In some cases, we were missing equipment, and I am telling you, those laboratories we were using are like the ones I have dreamed, and still we were missing things. For example, the VCL2 cabinets, we did not have them because we did not create those labs as biosafety labs, so we had to get the cabins” *(Leader of laboratory at a public university, Bogotá, SH-B-030).

 A fourth barrier perceived by several participants was the lack of clear mechanisms of communication between health secretariats, a national health institute, and laboratories from universities. For a public university in Bogotá, turning one of their laboratories into a diagnostic laboratory for COVID-19 to support the city´s health secretariat had to be put to a halt as the operation became financially unsustainable. As described by a university laboratory leader, the main problem for not being able to sustain the project was due to miscommunication, things that were not clarified from the beginning, and, again, resources that were not provided.

*“The flow of information was not as fluid as one would have liked. (…) and this lack of communication later derived in that we would find out late that we could not by any reason sell our service to other health providers. We depended 100% on the Secretariat of Health. And we had spent 1500 million pesos (approx. 435.0000 US dollars), a public university where each day is given less money. So, what could we do not maintain the service? (…) At the beginning in our meetings with the mayor’s office we were promised that we were going to receive reagents, supplies and equipment. So, we started. But then, they never came. Neither did they contribute to pay our personnel. After some fights, they sent us the reagents” *(Leader of a laboratory of a public university, Bogota, SH-B-030).

 In this case, it became clear that not all universities had the same capabilities for adjusting their work to the needs of local authorities, and furthermore, their ongoing involvement depended on sometimes precarious funding. Overall, academics had to quickly adapt to a complex landscape of intersectoral collaboration characterised by ongoing demands for useful and timely information, coupled with uncertainty over how to present such evidence and coordinate their actions with health system partners.

###  Private Sector

####  Roles 

 The main roles played by the private sector were: (1) economic (by providing large donations to support hospital capacity including availability of beds), (2) strategic (coordinating and mediating intersectoral relationships), and (3) logistic (by lending their networks and know-how to produce COVID-19 related supplies that were in high demand) (Table 4).

**Table 4:** Private Sector Roles

**Role**	**Actions**	**Outcome**	**Examples**
Economic	Donations of PPE, ventilators, hospital beds, testing supplies, drugs, medical oxygen and cost of training for managing critical care patients	Support expansion of clinical care capacity locally	A large space dedicated to commercial activities was transformed into a transitory hospital to attend non-COVID-19 patients. It hosted 274 beds for low and middle complexity and was dismantled after five months of operation (Bogotá) A non-for-profit development agency collected approximately 6 million dollars to install 149 intensive care units. A total of 170 000 PPE was delivered to the public and private hospital network (Bogotá) An important private association donated to the University of Cartagena a robot to process large number of samples in its laboratory (Cartagena)
Strategic	Alliances with other organizations Implementation of periodic intersectoral committees Brokering relationships	Support decision-making Establish consensus on public health actions Promote intersectoral coordination by tackling common issues	The chambers of commerce in each city set up intersectoral tables to support decision-making and allocate responsibilities among key actors A non-profit development agency in Bogotá and Cali that brings in international cooperation resources and has close ties with national donors became a key actor in supporting local governments
Logistic	Providing access to networks and supply chain "know-how"	Involvement of key actors in production chains Optimization of logistic services	Textile industry supported national health bodies in the manufacture of facemasks and PPE to supply healthcare providers (Cartagena) Animal feed industry provided gelatine from bovine for producing antifungal or antimicrobial required to PCR tests (Bogotá) Non-for-profit development agency provided logistics for delivery of foodstuffs to vulnerable population (Cali)

Abbreviations: COVID-19, coronavirus disease 2019; PPE, personal protective equipment, PCR, polymerase chain reaction.

####  Coordination Enablers and Barriers 

 The first enabler for coordination, as exemplified by local business associations, were long-standing or pre-existing relationships among key private sector actors and economic leaders which were mobilized in response to the pandemic. Prior to COVID-19’s arrival in Colombia, chambers of commerce of Bogotá and Cali had already established “health clusters,” both in 2014, to encourage intersectoral collaboration on health system priorities. Cartagena’s chamber of commerce established an intersectoral “roundtable” in April 2020 in response to the challenges posed by COVID-19 to their local health infrastructure. This situation enhanced the organization’s perceived legitimacy and facilitated positive interactions concerning COVID-19, as one interviewee stated:

*“(…) what makes it easier for us to participate in the discussion and generate some solutions or initiatives that can respond to some of the challenges posed by COVID is that we work closely with companies. (…) The cluster is not us, but the sum of companies. And we are close to the big health insurers in the city, the big hospitals, and public authorities. So, we have been trying to adapt our agenda to respond to the situation, as we support private companies to address their challenges to manage the pandemic (…) *(Representative, business association, Bogotá, SH-B-007).

 Second, some participants of the study identified as an enabler their neutral position, considering the diverse interests that actors of the health sector traditionally hold. This self-professed neutrality favoured their role as mediators. In the words of a participant:

*“(…) Our business association does not have any particular interest or privilege, and it does not represent specific interests of any of the groups it congregates. Then, it becomes a neutral forum to be able to discuss in a calm way the issues that concern the city” *(Representative, business association, Bogotá, SH-B-007).

 Moreover, this perceived neutrality contributed to gathering relevant actors in virtual spaces where direct and regular communication could take place. These spaces (committees, roundtables, and the like) were also deemed important for coordinating emerging activities related to COVID-19, making participants publicly accountable for each task, and were results-oriented. As one of our interviewees from Cartagena put it:

*“Articulating different actors has been the success factor. First, because key decision makers participated in all meetings, second, they had clear commitments, and third, they knew that when they come here to the meetings, they must show results. This increased their motivation to show results, because good results were recognized” *(Representative, business organization, Cartagena, SH-D-006).

 Third, in relation to the conditions posed by COVID-19 specifically, coordination with the private sector was enabled due to the alignment of public and private interests. For instance, the chambers of commerce were motivated to support the health system, based on awareness that any action on health would have an economic impact:

*“We as a chamber don’t have to get involved in the health issue and I always said it to the actors at the table: ‘look, my only objective here is to be a facilitator so that you can take the necessary actions and to improve health and activate the economy.’ That was the objective” *(Representative, business association, Cartagena, SH-D-006).

 Investments towards the health sector needed to be agreed upon and coordinated. The participation of actors from the private sector in governmental forums for decision-making allowed direct involvement in the definition of investments and joint public-private actions, and therefore greater alignment between public and private interests:

*“In Cartagena, we worked hand in hand in a health roundtable in which the chamber of commerce, the mayor’s office, the government and us were present. Initially, it was the one that allowed, for example, to define investment for the sector, to be able to say that the investments were in beds, ventilators, vaccines, biosafety elements, it was a joint effort, the foundation was part of that working group and it took a leadership role in achieving that there was, precisely, consensus” *(Representative, non-profit foundation, Cartagena, SH-D-018).

 Under the circumstances of urgency posed by COVID-19, private organizations’ networks contributed to establish further alliances. As one study participant reported, by fulfilling a role as funders, the private sector not only contributed with economic resources but also with a network of actors that came with different capabilities. In this way, some organizations that have worked in the past in the establishment of alliances gained a strategic position to collaborate with others to respond to challenges:

*“We made an alliance with a market chain to facilitate the delivery of groceries to vulnerable population, because they were the ones who had a better network to access the products. At this time, and it happened everywhere, many actors we knew came to us and say: ‘I can do this,’ and based on what we could do, we worked with them for the better” *(Representative, private development agency, Cartagena, SH-D-029).

 A participant also indicated that he had witnessed how their organization had transitioned from a philanthropic organization that provides donations to an organization that has a role in shaping local agendas, acquiring further political power:

*“We were focused on supporting economically governmental programs such as housing projects, youth programs, etc. But our support was mainly economic. But nowadays, we went from being just donors to influence local agendas” *(Representative, non-profit foundation, Cartagena, SH-D-018).

 Fulfilling these roles also came with major challenges. The first perceived barrier for coordination was the lack of trust among different stakeholders (eg, between health providers and health insurance companies, between health insurance companies and local authorities, between government agencies and health secretariats and long-standing tensions between actors from the local health system due to scarce resources or mismanagement). This long-standing situation is seen as a hindrance that operates at many levels: individual, organizational, systemic:

*“The lack of trust among health providers, health insurance companies, and local authorities, has been a major limitation for the health sector, even though we have improved this in the course of the pandemic. The lack of trust among sectors is a limiting factor for making actions effective, and there is still a fundamental problem in this regard” *(Representative Ministry of Health, SH-A-019).

 While it is not possible to resolve long-standing issues of mistrust in the context of the pandemic, the fact that acting on COVID-19 represented a common purpose (with direct consequences for everyone involved) helped with providing a more trusting environment for planning and delivery of public health action. However, from the narrative of the participants, it is not clear how issues of trust were negotiated in practice. Participants only stressed that these tensions had to be addressed first if effective actions against the virus were to be implemented.

 The second barrier to intersectoral coordination was a perceived lack of leadership from local government, particularly at the beginning of the pandemic, as a representative of national level body argued:

*“...the answer has to do with leadership. And local leadership, of being able to put different forces to speak, including the community of a department for example, or of a big city, and put people to point towards the same goal, that’s success” *(Representative, national institute for health, SH-A-018).

 In Cartagena, for instance, it was reported that from the onset of the virus, different actors were disorganized and there was a momentary power vacuum due to the transition of local leaders. With new administrations, accumulated debts, and a high contagion rate, Cartagena became a focus for intervention by national government. There was a designated coordinator from the national health ministry to organize stakeholders in the region and ensure effective actions to mitigate the spread of the virus and mortality rate of the disease. The contribution of different stakeholders was facilitated by plans coming from the Ministry of Health that underscored the importance of adaptation and availability of resources.

 One participant mentioned the need for a space where horizontal leadership could take place, rather than the promotion of single leaders. While strong and engaged leadership is beneficial for intersectoral coordination, he stressed that to mediate between different actors it is necessary that everyone contributed in what he called “collective leadership:”

*“That’s why I’m talking about collective leadership, because sometimes the person feels like a leader: I don’t need anyone, I just do everything... but no, this is not today’s case. It is easy to call for teamwork, but very difficult to apply it. There was a lack of leadership in the city because there was no one to articulate them and sit them all down and I believe that we as a Chamber had that ability to seat them all at one table” *(Representative, business association, Cartagena, SH-D-006).

 A third barrier signalled by some participants was self-interested behaviour. The relationships established between the private sector, local authorities, and public administrators differ depending on who is doing the ruling. There are differences in governing structures and styles at diverse levels: city, departmental, and national. It is clear for private sector actors that there cannot be a standardized coordination strategy. This is something that should be negotiated with every local leader. But, to some participants, resistance to leadership can become a barrier for coordination when “egotism” and “individualism” undermine collective action:

*“There were undoubtedly leadership problems. Why? Due to egotism. Here in Cartagena, we suffer from big egos and individualism. But these egos (of a mayor, a governor, a congressman, an insurance company, etc.,) prevent us for moving forward. With COVID-19, people started leaving their egos aside” *(Representative, business association, Cartagena, SH-D-006).

## Discussion and Conclusion

 Motivating the involvement of adjacent sectors in public health actions has proven challenging.^31^ Our study highlighted the roles that universities and the private sector played in response to COVID-19. These included augmenting Colombia’s clinical capacity and laboratory testing capacity, providing evidence to inform public health decision-making in response to unfolding events, coordinating actors from different sectors to address common issues, and extending networks and skills to support actions for community reach, technology development, and logistics.

 Even though Bogotá, Cali, and Cartagena differ in their population, the size of their territory, and in their installed network of health providers and universities (with Bogotá the largest and Cartagena the smallest of the three), we found that in the three cases academic institutions and the private sector played similar roles. Differences among the cases were noted primarily in styles of local government, community responses and contagion rates, as policy-making on COVID-19 was decentralized to each department.

 The roles played by academia and the private sector were underpinned by varied sectoral motivations. Firstly, both the academic and private sector were prompt to act in solidarity as the “right thing to do” given the strain on the healthcare system. This intrinsic motivation mirrors the concept of COVID-19 as a stimulus for collaboration found in relation to other health systems.^32^ Given that COVID-19 represented an outbreak of a novel and potentially lethal contagious disease, it is not certain that such motivation for collaboration would be applicable in relation to other public health problems unless an equally pressing need is demonstrated. Secondly, given the major economic repercussion of extensive lockdown measures taken to contain the virus, both sectors were motivated to help the health system so the economy could reopen as soon as possible. This was a primary motivation for the private sector in representation of local companies. The acute impact of COVID-19 on health and the economy may also be a motivating factor specific to this disease. One lesson from the response to COVID-19 for other areas of public health is to support the construction of evidence to motivate collaborative action by highlighting (*a*) the relevance of collaboration among sectors to tackle other public health problems and (*b*) linkages between health policy and other areas of the economy.^33^

 Thirdly, an underlying motivation for the intervention of both sectors in the response to COVID-19 in the country, was that their actions could amount to further recognition and reputational legitimacy. In addition to a sense of commitment, there was competition within and among private organizations and universities for responding to the crisis using research, resources, infrastructure, or skills. As others have pointed out, there is an established correlation between charitable activities and future financial performance as well as improved relations with local authorities.^34^ For academia, COVID-19 may have represented an opportunity to demonstrate their relevance and impact to government entities as an influential stakeholder concerning the university sector’s role in the field of science and evidence-based policy-making. For the private sector, COVID-19 could also represent an opportunity for increasing involvement in public local affairs.

 This qualitative study focussed on initial *processes* of intersectoral collaboration in response to COVID-19; further studies (eg, longitudinal, cross-sectional, surveys) are needed to evaluate *outcomes* of such collaboration, including: its sustainability beyond the initial response to the pandemic; potential positive effects (eg, improved prioritization in decision-making that reflects different sectoral needs); and possible unintended consequences (eg, perverse economic incentives associated with deepening private sector involvement in health service planning and delivery).

 Barriers and enablers to academia and private sector involvement stemmed both from pre-existing conditions of the Colombian health system (substantive) and those that emerged in the time of crisis characterised by COVID-19 (situational). Substantive enablers for the involvement of both sectors were established infrastructure and fundraising capacity, pre-existing relationships with key actors (organizations and individuals), and alleged neutrality to fulfil a “brokering” role. Organizations with stronger institutional embedding had the agility to engage in the intersectoral response to COVID-19 (eg, some universities were able to move more rapidly into COVID-19 testing supported by their existing laboratory infrastructure, administrative facilities and access to finance). Substantive barriers to intersectoral coordination were onerous legal and administrative demands for certification of laboratories, pre-existing tensions and mistrust among healthcare actors, local authorities, and limited financial and human resources for scientific research.

 Situational enablers of intersectoral collaboration that emerged in response to COVID-19 were a lack of and pressure for needed evidence, the establishment of frequent communication channels and spaces for discussion, alignment of private and public interests such as pressure for re-opening the economy and need of supporting public health actions for this purpose, and government support to involve both sectors in processes of decision-making. Situational or emergent barriers identified were difficulty of access to supplies given the global demand, the urgency of action that accentuated differences between time needed for research and time for decision-making, problems of miscommunication in the middle of a crisis, and weak and diffused local leadership.

 Intersectoral coordination is influenced by past experiences, favourable starting conditions, and existing partnerships.^11,35,36^ While participants from our study also indicated the importance of pre-existing conditions and relationships, or substantive enablers, for supporting intersectoral actions, we found that the context of emergency posed by COVID-19 progressively generated emergent or situational pressures for intersectoral collaboration inside local health systems. Studies of initial stages of the pandemic in other countries have highlighted that the pandemic exacerbated coordination challenges, particularly between levels of government or different nations. In Brazil, differences in positions on how to control the pandemic intensified between the federal, state, and municipal governments, as well as between governments and the scientific community.^37^ Similarly, coordination efforts in the response to the crisis have been difficult to achieve among member states of the European Union as national responses to COVID-19 reflect different national preferences and political measures.^23^ In Colombia, discrepancies between levels of government have also had a significant impact on coordination efforts.

 Substantive and situational factors are not independent from one another. On the contrary, it appears that for effective intersectoral coordination they have to aid one another. For example, the formation of committees bringing together different stakeholders, in which both sectors actively participated, were perceived as spaces fundamental for coordinating intersectoral action. These committees that met regularly were not something new, but drew on substantive pre-existing resources (eg, health clusters previously consolidated, stakeholders’ networks). The innovative aspect of their execution that was accelerated because of the pandemic was both their regularity (eased by virtual platforms) and diversity (with the inclusion of new actors including public health scholars, epidemiologists, geographers, data managers and representatives of chains of production). These spaces were not only important for discussion and direct communication but also helped to strengthen ties between different organizations, building new forms of trust and shared accountability for delivering results. Moreover, in these spaces, people and organizations from the private sector acted as “boundary spanners,”^38^ bringing together stakeholders that had varied roles and interests in the implementation of actions in response to COVID-19.

 If the crisis enhanced the visibility of public goods and services that require uniting all sectors,^39^ it is important to reflect on the relationship between substantial and situational enablers and barriers for intersectoral work. That is, how substantial and situational enablers and barriers might interact and change one another. As others have suggested and COVID-19 has made clear, there is not a one-size-fits-all approach to intersectoral collaboration as relationships depend on diverse and evolving contexts.^40^ The scale and pace of the pandemic made intersectoral coordination a necessity, contributing to a more notable participation of academic institutions and private enterprise in the country´s response to COVID-19. But the emergence of these coordination processes depended as much on collective will and effort towards a common goal (pre-figured in this case by the conditions of the disease), as on pre-existing resources and relationships that are known to favour appropriate forms of coordination.

###  Policy and Practice Implications

 Intersectoral coordination implies bringing together multiple and distributed stakeholders, often with divergent interests. Our findings suggest that health policy effort should focus on developing shared substantive resources, as this will influence the situational resources that emerge in response to a crisis. We recommend encouraging investment in science and technology collaborations or partnerships (eg, between health systems, universities, and private enterprise) to develop and address local priorities for health systems research and, through such partnerships, improve their capacity to engage in intersectoral activity (eg, understanding their respective skills and expertise, and rhythms of working). More systematic models for enabling collaboration that could be adapted for the Colombian context already exist in other countries, such as the regional Applied Research Centres in England,^41^ which provide an organizational infrastructure for articulating and addressing local health system priorities through intersectoral collaboration.

 The development of situational resources in response to COVID-19 also provides lessons for developing substantive resources for intersectoral collaboration. Situational responses to COVID-19 indicated that pre-existing tensions between key stakeholders need to be addressed. Support from organizations willing to act as mediators between the health sector and other sectors can be of great relevance to cope with pre-existing social barriers for intersectoral work such as the lack of trust between actors, communication difficulties, problems with accountability, and weak or thwarted leadership. These social processes are mechanisms that have been identified to uphold the effectiveness of intersectoral partnerships and are a significant part of partnership functioning. Policy-makers should consider that these mechanisms are cyclical, that is, the more practical activities that are accomplished through partnership the more trust, communication and accountability is reinforced, and vice versa. With the contribution of “brokering organizations,” there should be a sustained effort to solidify and build trust, communication, and accountability early on, paving the way for deepening partnership activities later.

 Policy-makers and regulators need to act on both substantive and situational enablers for intersectoral coordination and consider preventing the perpetuation of power structures and status quo by including new actors and their competencies in decision-making spaces.^40^ We found that the crisis enhanced relationships between diverse actors and that previous and new spaces for collaboration turned into pivotal platforms that facilitated communication between them, aligned their interests, and brokered discussion on health priorities and how to tackle them. To sustain intersectoral collaboration in Colombia, and not revert to fragmentation which characterised the pre-COVID health system, we recommend that those formal and informal relationships built with universities as well as with the private sector continue to be encouraged and stimulated through “hard” mechanisms. These should include formal agreements, governmental incentives, and the creation of collaborative structures for developing shared priorities in the longer-term, acknowledging barriers and facilitators for meeting those priorities, and providing a durable space for regular intersectoral meetings and communication for delivering agreed priorities and monitoring progress.

###  Limitations and Further Study 

 Our analysis assessed collaboration among some actors within different economic sectors in response to COVID-19, but did not examine collaboration among types of government agency (for example, how health-related agencies worked with other government authorities, including migration and transport). Further research could address this gap by analysing relationships across government, in recognition of the roles of a variety of agencies in supporting the public health response. Other research has considered such relationships at the local level^42^ but further research is needed on the coordination of national level responses.^32,43^

 Our study only focussed on senior level stakeholders. Perspectives and experiences of intersectoral collaboration from medium and lower-level roles within universities, the private sector, and health system have been neglected. For example, it could be that senior representatives give a positive spin on their roles in response to COVID-19 to maintain their organization’s legitimacy. Further qualitative research is needed that includes the perspective of frontline staff on intersectoral working in response to COVID-19. Such work might examine the potential impact on the wellbeing of rank-and-file staff in universities associated with responding to COVID-19, to see how this compares with the impact on those involved on the front-line in the delivery of health services, for example, stress and burnout in both sectors has been reported.^44-46^ Observation of practices of intersectoral activity could not be undertaken, as collecting primary data needed to respect physical distancing measures. As we only conducted online interviews, the study reflected narration through actors’ voices, which can be considered as a limitation because the actors’ accounts have not been triangulated with other data (eg, observations of meetings). Online observational work could have helped to qualify some propositions made by the actors, for instance, the alleged neutrality of the “brokering role” in roundtable meetings. To qualify the actors’ accounts, it is important in future studies to conduct ethnographic work, and quantitative studies, to define and measure how intersectoral coordination influences health system planning and delivery.

## Acknowledgements

 We wish to thank the interviewees who participated in the study with anonymity.

## Ethical issues

 Ethics approval for this research was received from the institutional review board at Universidad de los Andes. The research was classified as minimal risk.

## Competing interests

 Authors declare that they have no competing interests.

## Authors’ contributions

 ST and AU designed the study. AU, NN, and VVG contributed to data collection. All authors contributed to analysis and interpretation, and reviewing and editing the manuscript. All authors had full access to all the data in this study and take responsibility for the integrity of the data and the accuracy of the data analysis.

## Funding

 This project was funded by the Colombian Ministry of Science, Technology and Innovation. Project code 1204101577001.
